# Correlating Hydration of Alkali-Activated Slag Modified by Organic Additives to the Evolution of Its Properties

**DOI:** 10.3390/ma16051908

**Published:** 2023-02-25

**Authors:** Vlastimil Bílek, Lukáš Kalina, Richard Dvořák, Radoslav Novotný, Jiří Švec, Jiří Másilko, František Šoukal

**Affiliations:** 1 Institute of Materials Science, Faculty of Chemistry, Brno University of Technology, 612 00 Brno, Czech Republic; 2 Institute of Physics, Faculty of Civil Engineering, Brno University of Technology, 612 00 Brno, Czech Republic

**Keywords:** alkali-activated slag, calorimetry, microstructure, retardation, pore solution, organic admixture, color change, shrinkage, setting

## Abstract

This study investigates the relationships between the hydration kinetics of waterglass-activated slag and the development of its physical-mechanical properties, as well as its color change. To modify the calorimetric response of alkali-activated slag, hexylene glycol was selected from various alcohols for in-depth experiments. In presence of hexylene glycol, the formation of initial reaction products was restricted to the slag surface, which drastically slowed down the further consumption of dissolved species and slag dissolution and consequently delayed the bulk hydration of the waterglass-activated slag by several days. This allowed to show that the corresponding calorimetric peak is directly related to the rapid evolution of the microstructure and physical-mechanical parameters and to the onset of a blue/green color change recorded as a time-lapse video. Workability loss was correlated with the first half of the second calorimetric peak, while the most rapid increase in strengths and autogenous shrinkage was related to the third calorimetric peak. Ultrasonic pulse velocity increased considerably during both the second and third calorimetric peak. Despite the modified morphology of the initial reaction products, the prolonged induction period, and the slightly reduced degree of hydration induced by hexylene glycol, the overall mechanism of alkaline activation remained unchanged in the long-term perspective. It was hypothesized that the main issue of the use of organic admixtures in alkali-activated systems is the destabilizing effect of these admixtures on soluble silicates introduced into the system with an activator.

## 1. Introduction

Alkali-activated slag (AAS) belongs to the wide and interesting group of alkali-activated materials (AAMs) and has a considerable potential to contribute to the sustainability of construction materials [1]. For the alkaline activation of slag or other suitable aluminosilicates, many types of activator [2] and their combinations can be used at various doses or concentrations, resulting in practically unlimited complexity of materials based on AAS. This, on the one hand, enables tailoring of AAS properties as required, but on the other hand, results in fragmented research with sometimes contradictory or unclear results. Therefore, it is necessary to look for the relationships between the measured properties and to be able to estimate them from simple observations.

In general, the course of alkaline activation consists of several stages, of which the starting stage is the dissolution of the glassy phase. Song and Jennings [3] stated that a pH greater than 11.5 is necessary for effective activation, as slag below this value does not dissolve effectively. During the slag hydrolysis, Ca^2+^ and Mg^2+^ are preferentially released into the solution followed by aluminate species and silicates [4]. After sufficiently high concentrations are reached, new hydration products of lower solubility than the original slag are formed. The main one is amorphous or poorly crystalline partially cross-linked calcium-aluminate-silicate-hydrate (C-A-S-H), sometimes also written as C-N-A-S-H due to the presence of an alkali (e.g., sodium) cation that balances the negative charge of tetrahedral aluminum [5]. C-A-S-H is the main strength-giving phase in AAS, but a wide range of other products can be formed, such as hydrotalcite, strätlingite, and other aluminates [4,5,6].

The type and quantity of hydration products depend on several factors, such as slag and activator composition, activator dose or concentration, and temperature. Powerful tools for monitoring the hydration kinetics are isothermal calorimeters. In the 1990s, Shi and Day [7] described basic types of heat flow curves depending on the activator nature and temperature and described stages of hydration similar to those for Portland cement hydration, i.e., the pre-induction period, the induction or dormant period, the period of accelerated and decelerated hydration, and further slowing down diffusion- or phase boundary-controlled stage [8]. For slag activation with NaOH, two peaks can be distinguished on the heat flow curve. The first one that occurs in the pre-induction period is related to the wetting and initial reactions between activator and slag grains, and after no or very short induction period the second peak that is associated with the formation of C-A-S-H takes place [4,7,9]. For activation with Na_2_CO_3_ and liquid sodium silicates (waterglasses), a new additional initial peak commonly emerges in the pre-induction period due to the reactions of anionic groups of these activators. After this peak, both of these activators exhibit a significant induction period, after which the third peak of acceleration/deceleration hydration occurs, showing the formation of a C-A-S-H gel [7,10,11].

In addition to the composition of the activator and precursor or temperature [9,12], the reaction process of AAS can be considerably affected by various organic additives. From this wide group, superplasticizers are the most studied in order to control the rheology [13]. However, other additives such as shrinkage-reducing admixtures (SRAs) deserve attention; not only to deal with the extensive shrinkage of AAS [14,15], but also due to their great effect on the hydration process [16,17,18,19,20]. This is inherently related to changes in the evolution of the microstructure and properties. For example, the main calorimetric peak has been reported to be associated with the highest rate autogenous shrinkage [21,22]. In addition to engineering properties, the famous color change of not only AAS but also ordinary concretes containing slag is well-known. It is believed to be somehow related to the reaction of slag in these systems [23,24].

However, a comprehensive overview of the relationships between the AAS hydration rate and the evolution of its properties from the fresh to well-hardened state supported by investigations of phase assemblage and microstructure, and its color change as well, is missing. Consequently, this is the main objective of this paper. Because different stages of AAS hydration usually more or less overlap, it is necessary to separate them to clearly observe the associated effects. Unlike other papers, in which SRAs are applied to reduce AAS shrinkage [25,26,27], we used various organic substances, and particularly hexylene glycol as a component of commercially available SRAs, to intentionally slow the AAS hydration process and separate its different stages. In addition, its role in AAS hydration is briefly addressed.

## 2. Materials and Methods

### 2.1. Materials and Sample Preparation

Ground granulated blast furnace slag with a Blaine fineness of about 400 m^2^/kg was used. According to the X-ray diffraction method with Rietveld analysis using Empyrean diffractometer (Malvern Panalytical Ltd., Malvern, UK), it consisted mainly of an amorphous phase (~87 wt. %) and small amounts of calcite, merwinite, and akermanite. Calcium fluoride was used as an internal standard. Its chemical composition determined using X-ray fluorescence is given in Table 1. Slag was alkali-activated by waterglass containing 16.1 wt. % Na_2_O and 30.7 wt. % SiO_2_ (silicate modulus of approximately 2.0) diluted with water to prepare the stock solution prior to mixing.

The dose of stock waterglass solution was adjusted to 4% Na_2_O with respect to the weight of the slag and the water-to-slag ratio (including the water from the original waterglass) was 0.35. Such plain AAS paste was modified by hexylene glycol (2-methyl-2,4-pentanediol) at the doses of 0.5, 1.0 and 2.0% with respect to the slag weight. The hexylene glycol dose was used for designation of the pastes, i.e., Hex-0 for the plain AAS paste without hexylene glycol, while Hex-0.5, Hex-1.0, and Hex-2.0 were used for pastes containing hexylene glycol. Hexylene glycol was added to the activating solution and briefly homogenized just before mixing the AAS paste.

The mixing of the pastes was carried out using a common hand mixer and a beaker in an air-conditioned laboratory at 25 °C. The time of adding the slag to the activating solution was taken as time zero, after which the mixing process started. The total mixing time was three minutes and consisted of four intervals: First 60 s of slow mixing, then 30 s at maximum mixing rate, 30 s rest for scraping the paste down into the batch, and finally 60 s at maximum mixing rate. The pastes prepared in this manner were then used for the tests described in the following sections.

### 2.2. Methods

#### 2.2.1. Isothermal Heat Conduction Calorimetry

Hydration of AAS was monitored using a TAM Air 8-channel calorimeter (TA Instruments) at 25 °C for 28 days. For the measurement, the amount of paste corresponding to 4.0 g of slag was placed in a 20 mL PE vial, tightly sealed with a screw cap, and inserted into the calorimeter. Recording of heat flow started five minutes after the start of the mixing. The vials with water were used as a reference.

#### 2.2.2. Physical-Mechanical Testing

The initial and final setting time was tested using a common Vicat needle with a diameter of 1.13 mm. Measurement was carried out on the basis of EN 196-3 [28] but it was not made under water. Instead, the surface of the sample was covered with paraffin oil to prevent drying and ensure autogenous conditions, which were also used in other experiments. Furthermore, the pastes did not have normal consistency as is prescribed for cement testing but had the same composition as in all other experiments (Section 2.1), i.e., the water-to-slag ratio of 0.35 and the waterglass dose of 4% Na_2_O with respect to the slag weight.

Monitoring of changes in ultrasonic pulse velocity (UPV) during the ongoing hydration was carried out using a Vikasonic device (Schleibinger Geräte Teubert u. Greim GmbH, Buchbach, Germany) working with a frequency pulse of 54 kHz. The measuring cell had the same shape as the ring for the setting time determination using a Vicat needle (EN 196-3) with probes mounted in the lower and upper base of the path length of 40 mm. UPV was automatically calculated from the path length and transit time. The surfaces of the probes were coated with couplant grease and separation foil to prevent direct contact of the probes with the measured material. UPV values were recorded in one-minute intervals from the start of the measurement in the fresh state for as long as possible or until the signal was disturbed.

Autogenous dimensional changes were determined using the hydrostatic weighing method at 25 °C controlled by a circulating thermostat. The paste was filled into a polyurethane condom and then approximately 90 g of the paste was tied with a fishing line and hung on an analytical balance with readability of 0.1 mg. First, the specimen weight in air was recorded, then the specimen was immersed in paraffin oil and its weight was automatically recorded in one-minute intervals for at least 28 days. The relative volume change Δ*V* (in volume percent) was calculated using the Equation (1), where *m_t(ini)_* and *m_t_* are the weight of the specimen immersed in oil at the initial time to which shrinkage is related (the first value in oil in this work) and the weight at a given time of measurement, respectively, and *m_air_* is the weight of the sample before immersion into the oil.

(1) $$∆V=\frac{m_{t\left(ini\right)}-m_{t}}{m_{air}-m_{t\left(ini\right)}}$$

The compressive strength was determined on cubic specimens with nominal edge size of 25 mm using the DESTTEST 4310 Compact A (Beton System, s. r. o.). After mixing, the pastes were cast into the molds and sealed with polyethylene plastic bags for 23 h, after which they were demolded and tested to obtain 24-h compressive strength or sealed with stretch foil and stored in a chamber at 25 °C until the time of testing, i.e., 2, 3, 7, 10, 14, 21, and 28 days. At each age, 2 to 3 specimens of each paste’s composition were used.

#### 2.2.3. Pore Solution Composition

For the isolation of the pore solution, fresh AAS pastes were cast into the cylindrical plastic molds with a diameter of 52 mm and a height of 60 mm with inserted stretch foil to prevent them from drying and carbonation as well as to facilitate its demolding. The pore solution of the fresh pastes was obtained by the vacuum filtration using a Büchner funnel. Later, when the paste stiffened and set, the foil was removed from the samples and they were placed into a steel mold to press the pore solution out using the hydraulic device DESTTEST 4310 Compact A (Beton System, s.r.o., Brno, Czech Republic). The obtained pore solution was place in a syringe equipped with a nylon filter with a pore diameter of 0.45 μm and immediately diluted in a plastic volumetric flask using demineralized water. The concentration of the selected elements was determined by an optical emission spectrometer with inductively coupled plasma (ICP-OES) Horriba Jobin Yvone, type Ultima 2. The sample was transported using a peristaltic pump into a nebulizer and further carried by argon into a torch. The elemental concentration of the pore solution was automatically calculated on the basis of the calibration curves for standard solutions and the background represented by demineralized water.

#### 2.2.4. Assessment of Color Change

**Figure 1:**
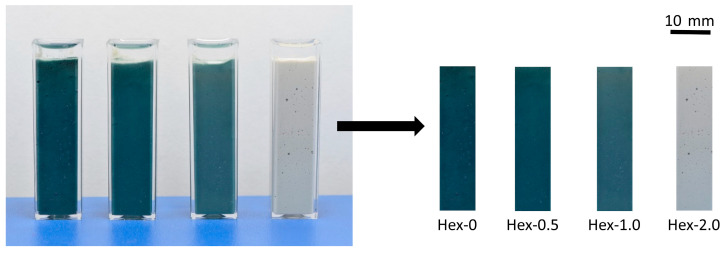
Illustrative example of a photograph captured after 7 days of hydration and extraction of the areas of samples for processing of the color change.

To record a well-known but still not fully understood blue/green color change in AAS pastes during hydration, samples of the pastes containing 0, 0.5, 1, and 2% of hexylene glycol were filled into the plastic cuvettes with a square cross-section of 10 mm × 10 mm and a height of 50 mm. To prevent drying and air reactions, the surfaces were covered with a paraffin oil. Photos of these AAS paste-containing cuvettes were continuously captured for 28 days every 30 min using a digital camera equipped with a macro lens and connected to a computer. This experiment was carried out in an air-conditioned room at 25 °C. The obtained photographs were then automatically processed in MATLAB software to separate out only the areas of the samples as illustrated in Figure 1. Each cropped area of photo was then divided to red, green, and blue channel matrix. For each picture an average value of each channel was calculated. Because the maximum intensity of a pixel in each channel matrix has an integer of 255, we use inverted value. Subsequently, the color change in time was simply demonstrated as the paste darkness calculated as the sum of the inverted integers for red, green, and blue. The extracted areas were also used to make a time-lapse video.

#### 2.2.5. Phase Assemblage

The microstructure and formation of the hydration products were assessed using scanning electron microscopy (SEM), thermogravimetric analysis (TGA), and X-ray powder diffraction (XRD). Before these analyses, the hydration reactions were stopped using the solvent exchange method by immersing the broken parts (SEM) and crushed paste (TGA, XRD) in the isopropyl alcohol and then drying at 40 °C. SEM was performed on both fracture surfaces and ionic polished samples embedded in the epoxy resin. Fracture surfaces were observed using EVO LS 10 (Carl Zeiss Nts, LCC, Peabody, MA, USA) with an accelerating voltage of 10 kV in the mode of secondary electrons, while polished surfaces were observed using JSM-7600F (JEOL Ltd., Tokyo, Japan) with an accelerating voltage of 5 kV in the mode of backscattered electrons. The TGA was carried out in air atmosphere with a flow rate of 100 mL/min with a temperature ramp of 10 °C/min from 40 to 990 °C using a Q600 SDT analyzer (TA Instruments, New Castle, DE, USA). XRD was carried out using Empyrean diffractometer (Malvern Panalytical Ltd., Malvern, UK) in the Bragg–Brentano configuration at the range of 5–90° 2θ with step of 0.013° 2θ. An X-ray tube with copper anode, a voltage of 40 kV, and a current of 30 mA was used. Data were evaluated using HighScore Plus software.

## 3. Results and Their Discussion

### 3.1. Overall Course of Hydration and Hydration Products

**Figure 2:**
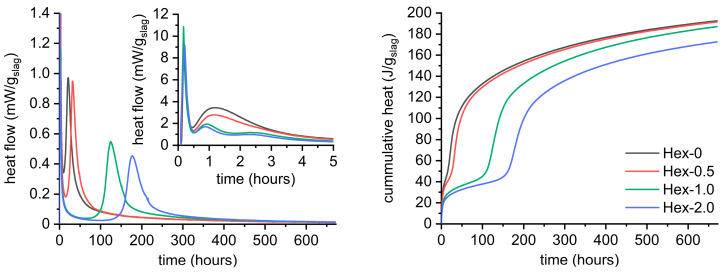
Calorimetric response of waterglass-activated slag containing 0–2% of hexylene glycol.

The effect of hexylene glycol dose on the AAS hydration is given in Figure 2. The reference paste without hexylene glycol exhibited a common type of calorimetric curve for waterglass-activated slag comprising three peaks, which is consistent with the literature [7,29,30]. The first of these peaks occurring within the first 30 min is associated with the initial contact of activating solution with slag grains, i.e., wetting and dissolution of the slag particles and external mixing. The second one, which reaches maximum at approximately one hour, was attributed to the gelation of silicates from activator due to dissolved species from slag [31,32,33]. This peak dramatically decreased with increasing dose of hexylene glycol. Finally, the third peak occurs, where extensive hydration takes place, resulting in the formation of the C-A-S-H and other hydrates, with a maximum heat flow at 21 h for the reference paste. However, the presence of hexylene glycol and many other organic additives strongly affected the timing of this ‘peak of the bulk hydration’ [31,32].

It is worth noting that similar effect can be reached by other various organic additives, as clearly illustrated in Appendix A (dose of 2%). The strongest effect of the substances used was observed for ethanol, which had the maximum of the third peak at approximately 410 h, or in other words approximately 20 times later when compared to the reference paste. Furthermore, Figure 2 and Appendix A show that the lower the gelation peak, the more delayed the bulk hydration peak.

**Figure 3:**
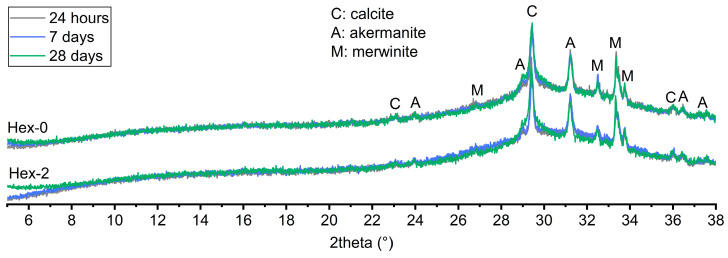
XRD patterns of waterglass-activated slag with 0 and 2% of hexylene glycol after 28 days of hydration.

**Figure 4:**
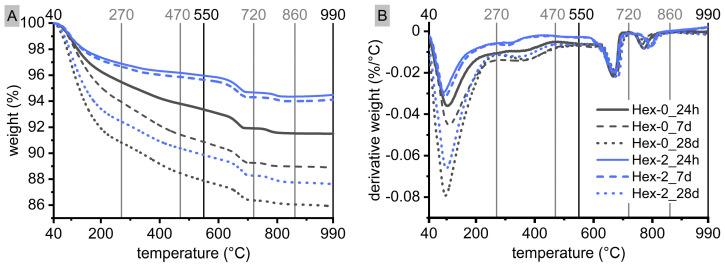
Changes in weight (**A**) and their temperature derivative (**B**) of waterglass-activated slag pastes with 0 and 2% of hexylene glycol during the first 28 days of hydration with highlighted ranges that were evaluated (see Table 2).

It should be noted that although hexylene glycol greatly retarded the hydration of AAS, it did not dramatically change its overall mechanism from the long-term perspective. This is suggested by the evolution in cumulative heat and further supported by XRD after 28 days (Figure 3) and by thermogravimetry after 24 h, 7 days, and 28 days (Figure 4). No crystalline phases formed during the hydration process were detected by XRD regardless of the presence of hexylene glycol. Only those originating from the raw slag, i.e., calcite, akermanite, and merwinite were identified (Figure 3). This corresponds to previous findings [34,35,36] that highly amorphous products are formed in waterglass-activated slag, mainly C-A-S-H, which is more crystalline in NaOH-activated slag.

The only notable difference in the XRD patters is a different intensity below 8° 2θ, i.e., in the region corresponding to the presence of C-A-S-H [37,38]. For the reference paste (Hex-0), the intensity only increases very slightly over time. On the contrary, the Hex-2 paste showed identically low intensities after 24 h and 7 days, followed by greatly increased intensities after 28 days. A slight change also related to the greatly increased amounts of C-A-S-H in Hex-2 paste after 28 days is also visible as an increased background between 29 and 30° 2θ. Unfortunately, the diffractions of C-A-S-H in this region [37,38] cannot be clearly seen due to the presence of akermanite and particularly calcite. In addition, the issue of poor visibility of C-A-S-H in these XRD outputs is its already mentioned highly amorphous structure. The different evolution of the XRD pattern for Hex-0 and Hex-2 paste corresponds to their different rate of reaction (Figure 2), because considerable amounts of C-A-S-H can be expected in Hex-0 paste even after 24 h, while the onset of the bulk hydration peak of Hex-2 paste occurred after one week.

Thermogravimetry (Figure 4, Table 2) confirmed a great effect of hexylene glycol on amounts of hydrates over time, as indicated by weight loss in the temperature range of 40–550 °C. It is mainly related to the dehydration and dehydroxylation of C-A-S-H, but some contribution of the hydrotalcite-like phase is also possible, particularly in the range of 270–470 °C [39,40,41]. For the reference paste, the amount of hydrates (40–550 °C) gradually increased over time. Distinctively different behavior was observed for Hex-2 paste, for which the weight loses of the Hex-2 paste almost did not changes between 24 h and 7 days, while greatly increased between 7 and 28 days. This correlates well with the retarding effect of hexylene glycol apparent from calorimetric outcomes (Figure 2).

Weight loss in the range of 550–990 °C was not significantly affected by hexylene glycol (Figure 4, Table 2). In this range, two different steps can be distinguished. The first took place between 600 and 700 °C and was related to the decomposition of calcite originating from slag, while the second region around 800 °C is usually assigned to the decomposition of C-A-S-H along with the crystallization of wollastonite [39,42]. Moreover, it is very likely to be affected by stopping hydration using the solvent exchange method because organic solvents greatly modify TGA outcomes in this temperature range also for Portland cement systems [43].

### 3.2. Correlations during the Gelation Stages of AAS Hydration

**Figure 5:**
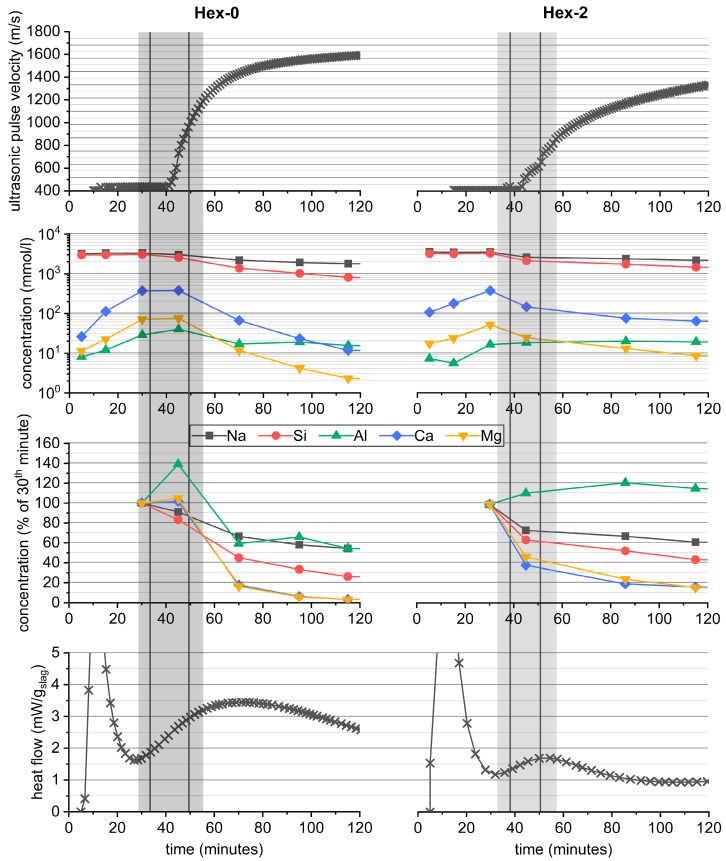
Correlations of the calorimetric response of waterglass-activated slag with its pore solution composition, the ultrasonic pulse velocity and the setting time (vertical lines show average values of the initial and final setting time, shaded area around them shows the setting period including the sample’s standard deviation).

Figure 5 correlates the already showed effect of hexylene glycol on the gelation peak (Figure 2) with the evolution of early mechanical properties represented by the Vicat needle test and UPV. In addition, the changes in the pore solution composition over time are also included.

Despite its great effect on AAS hydration, hexylene glycol did not have significant effects on initial and final setting times determined using the Vicat needle, as it remained around 35 and 55 min, respectively, regardless of the dose of hexylene glycol. At the same time, the onset of UPV was not affected by the presence of hexylene glycol, while its subsequent increase slowed slightly. All these phenomena correlated well with the pore solution composition, which was roughly the same for the Hex-0 and Hex-2 pastes during the first 30 min: The concentrations of Si and Na introduced into the system mainly within the activator remained around 3 M, while the concentrations of other elements originating from the dissolving slag, such as Ca, Mg, and Al, gradually increased. The maximum concentration of Ca was around 0.4 M irrespective of the presence of hexylene glycol, while the maximum concentrations of Mg and Al were lower by approximately one order of magnitude compared to those of Ca. After the first 30 to 45 min, the concentrations of all elements started to drop quite rapidly, particularly for the reference Hex-0 paste; and until the 118th minute, they decreased as much as by an order of magnitude compared to their maximum values. The determined values as well as the observed trend of the evolution of the pore solution composition over time correlate well with a recently published study [26], in which a slightly higher dose of waterglass (5% Na_2_O) with a somewhat lower silicate modulus (1.2) was used to activate the mixture of slag/fly (50/50).

In the case of the reference paste, the simultaneous sharp increase in mechanical properties and high concentrations of elements originating from slag in the pore solution after 30 to 45 min point to the concurrent formation of new solid products and the continuing dissolution of slag. At later stages, the dissolved species are consumed rapidly. Considerably slowed down consumption of dissolved species was observed for Hex-2 paste, which correlates with less heat generated at this stage and with a slightly slower increase in the UPV velocity.

**Figure 6:**
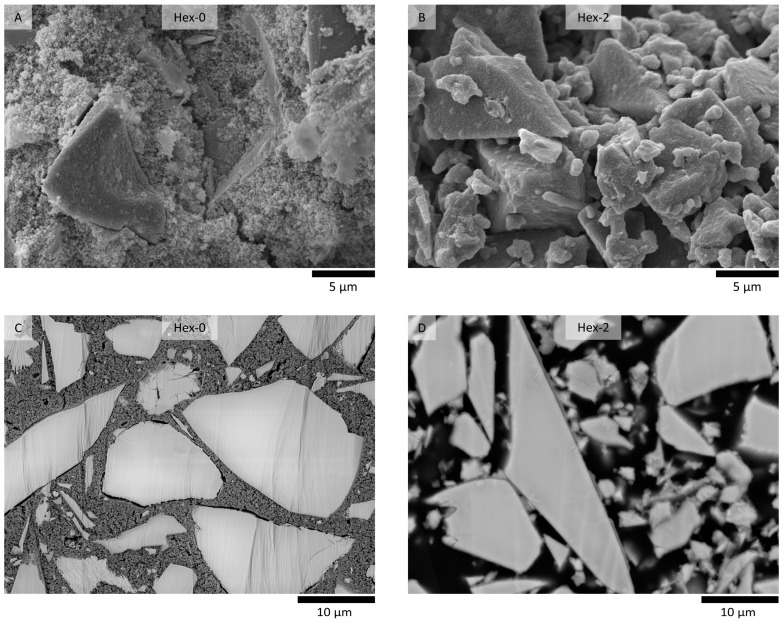
Microstructure of waterglass-activated slag pastes without hexylene glycol (**A**,**C**) and 2% of hexylene glycol (**B**,**D**) after 5 h of hydration observed as fracture surfaces (**A**,**B**) and after embedding in the epoxy resin and ionic polishing (**C**,**D**).

This behavior is likely related to the effect of hexylene glycol on microstructural evolution in these very early stages of reaction, as shown in Figure 6. During the first 5 h, a highly porous but otherwise homogeneous matrix of the initial reaction products was formed among the slag grains in the reference paste (Figure 6A,C). Note that a similar microstructure has already been observed by Sun et al. [44] and was attributed to the gelation of the activator, even if the slag was replaced by quartz, due to the preparation of the cryogenic sample. These similarities support the theory that the additional initial calorimetric peak of the waterglass-activated slag is related to the gelation of the activator [31,32].

In contrast to the reference, no bulk matrix can be found in the paste with 2% hexlyene glycol (Figure 6B,D). Instead, a thin layer of the reaction products covers the slag grains and binds them together through the adjacent surfaces, enough to indicate setting by the Vicat needle test or an increase in UPV. Simultaneously, it shows that the great reduction in the gelation peak does not lead to a significant prolongation of the workability period. To reach it, the gelation peak should be delayed, as in the case of citric acid [45].

**Figure 7:**
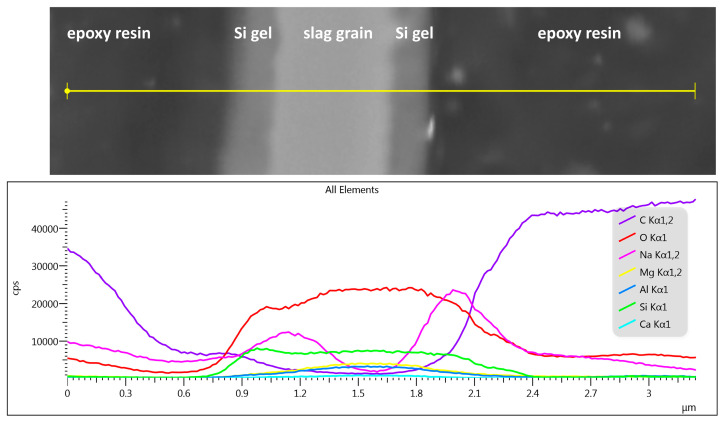
Line scan of the composition of waterglass-activated slag paste with 2% of hexylene glycol after 5 h of hydration embedded in an epoxy resin and ionically polished; accelerating voltage of 5 kV.

The darker gray shade of the formed layer compared to that of the slag particle means that the former is composed of lighter elements. The more detailed image given in Figure 7 reveals that the thickness of this layer can reach approximately 200 to 300 nm and is quite dense, although the resolution is limited. The line scan of the elemental composition given in the same figure showed that these products are rich in Na and Si, which confirms that the layer originates mainly from the gelation of the activator. The content of other elements such as Ca and Mg is also possible and, with respect to the evolution of the composition of the pore solution over time, even probable (see Figure 5), but their signal is limited by a low accelerating voltage.

In-depth investigations of the role of hexylene glycol during the gelation stages of AAS hydration are beyond the scope of this paper. Nevertheless, we propose that it is closely related to the action of the organic substances in the activating solution. In our previous article [33], the decreasing intensity of the gelation peak in AAS pastes in the presence of organic substances correlated with the acceleration effect of hexylene glycol on gelation of model systems. These were of two types: activating solution of waterglass gelled by calcium hydroxide and, to avoid the presence of calcium ions, silicate sol gelled by sodium chloride. The results obtained suggest that the organic admixtures destabilize the silicate species. In some cases, they even changed the structure of the arising gels. Their action could be related to their hygroscopicity and very limited miscibility with silicate solutions, but further research revealing their specific action is needed.

### 3.3. Correlations during the Bulk Hydration Stages of AAS Hydration

**Figure 8:**
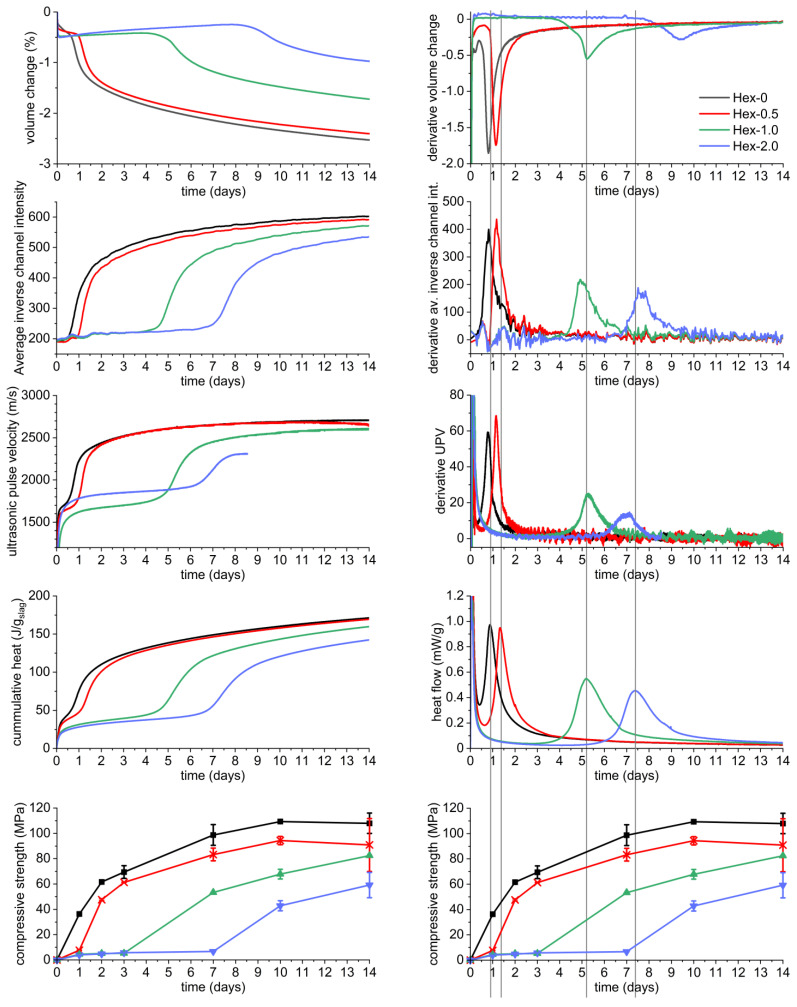
Correlations of physical-mechanical characteristics of waterglass-activated slag with its calorimetric response (vertical lines intersect the maximum of bulk hydration calorimetric peak).

The third calorimetric peak is mainly associated with the acceleration/deceleration stage of hydration [7,8], the main hydration peak [46], or the bulk hydration peak [31,32], associated with massive precipitation of reaction products, especially the C-A-S-H gel. This is in accordance with the results of thermogravimetry (Figure 4). Therefore, it can be expected that the occurrence of this peak is related to the evolution of the microstructure and properties. This is confirmed in Figure 8, which shows a perfect correlation of the calorimetric response with the development of the characteristics of AAS tested. These were autogenous shrinkage, compressive strength, UPV, and also color change. Note that the derivatives of these parameters even reflect the shape of the bulk hydration calorimetric peak.

The presented results of autogenous shrinkage in context with the bulk hydration peak on the calorimetric curves are in agreement with those of recent studies of Li et al. [21,22]. A similar but not as detailed effect of SRA on compressive strength development was observed in our previous study [17]. Note that the pastes can reach certain mechanical properties (compressive strengths of few megapascals) during the gelation stage, but their major increase is related to the bulk hydration peak. These issues further emphasize the need for adequate curing of AAS-based products prior their testing or practical use, because testing of immature sample can lead to misleading results.

The quality of curing can be partially indicated by the level of fascinating blue/green coloration of the slag-containing binders. However, it can be an aesthetic issue in many applications [23]. The phenomenon of intensification of the color with increasing curing time is well known, probably since the coloration was first observed. Figure 8 clearly reveals that it is intimately associated with the peak of bulk AAS hydration. In this figure, the coloration is numerically expressed as the darkening of the sample. For a better idea of the color change, the reader is kindly referred to Video S1 (Supplementary Materials), in which the color change over time is again correlated with the calorimetric response of AAS.

The exact origin of the coloration has not yet been fully understood. It is most often explained by the progressive oxidation of sulfide-based compounds present in slag under anoxic conditions to di-, tri-, and tetrasulfur radical anions [24,47]. Sulfide species are expected to be released from the glassy phase during its dissolution [6,24], which is in good agreement with the appearance of the bulk hydration peak in the present study.

The presented findings clearly show that the timing of the onset of the color change can be tailored by the nature and dose of an organic additive, which could be exploited for further investigations of this interesting phenomenon. In practical applications, the coloration indicates that the products are at least partially mature, able to be safely demolded, transported, etc. It should be emphasized that the absolute values of the strengths can be very different for different activators despite the similar color of the specimens due to the different nature of the reaction products [36].

## 4. Conclusions

This paper summarizes the relationships between the evolution of various properties and calorimetric responses of slag activated with waterglass with a relatively high silicate modulus (2.0) and an intermediate dose (4% Na_2_O with respect to the slag weight), modified by varying the dose and type of organic admixture, especially hexylene glycol. Based on the results presented in this paper and their discussion with the literature, the following conclusions can be drawn:

- Hexylene glycol and many other organic admixtures significantly affected the hydration process of AAS by means of decreased intensity of the gelation peak and simultaneously prolonged induction period, i.e., retardation of the bulk hydration peak.
- The position of the second peak is crucial for the timing of setting or loss of workability, while the intensity of this peak is related to the initial compressive strengths that reach a few to several MPa. After that, the hydration goes into the induction period of minimized heat flow and can last several days or even weeks depending on the type and dose of the organic admixture.
- The bulk hydration peak perfectly correlates with a great increase in strength, autogenous shrinkage, and ultrasonic pulse velocity, as well as with the onset of a fascinating color change from off-white to green/blue, characteristic for AAS. Therefore, the color change is a very simple and practical indicator of the bulk hydration of the slag and thus tells something about the maturity of the material, particularly under anoxic conditions.
- The influence of organic additives lies in their action during the gelation stages of hydration. Hexylene glycol caused the formation of a few hundred nanometer thick layer of silicate-based gel on the slag surface instead of the formation of the continuous matrix in the bulk of the interstitial space, typical for waterglass-activated slag without additives. This seems to be the main reason for both the decreased rate of slag dissolution and the decrease in the formation of reaction products, resulting in a prolonged induction period.
- Despite the strong retardation effect of hexylene glycol on AAS hydration, the overall reaction process did not change distinctively. No new crystalline phase nor the significant changes observed by XRD and thermogravimetry, respectively. The latter only confirmed the retardation effect by very low and almost the same weight losses of the paste with hexylene glycol after 24 h and 7 days.

The physical and chemical changes over the first weeks of AAS hydration were comprehensively summarized. This allows estimations of the behavior of from the very early setting stage to long-term hardening stage based on its calorimetric response or potentially based on the evolution internal temperature. Although the changes in hydration rate were intentionally carried out using intentional addition of hexylene glycol, further research on the interactions between organic substances and AAS is necessary, especially during the gelation stage, to fully understand the observed phenomena. This would promote the tailoring of organic admixtures for alkali-activated systems. At the same time, this paper can serve as a starting point for an in-depth study of the origin of color change and other not fully understood phenomena related to the hydration of AAS.

## Figures and Tables

**Table 1:** Chemical composition of the used slag determined using X-ray fluorescence.

SiO_2_	Al_2_O_3_	Fe_2_O_3_	CaO	MgO	SO_3_	K_2_O	Na_2_O	P_2_O_5_	TiO_2_	Mn_2_O_3_	SrO	ZnO	Cl	LOI
37.2	9.32	0.27	39.1	9.50	1.53	0.43	0.40	0.02	0.03	0.61	0.06	0.02	0.03	1.24

**Table 2:** Weight losses (%) of waterglass-activated slag pastes with 0 and 2% of hexylene glycol evaluated in various temperature ranges.

Sample	40–550 °C	270–470 °C	550–720 °C	720–860 °C	550–990 °C	40–990 °C
Hex-0: 24 h	6.64	1.67	1.45	0.39	1.87	8.51
Hex-0: 7 d	9.13	2.50	1.61	0.28	1.98	11.1
Hex-0: 28 d	12.1	2.36	1.51	0.32	1.97	14.1
Hex-2: 24 h	4.03	0.71	1.30	0.31	1.48	5.51
Hex-2: 7 d	4.35	0.80	1.34	0.31	1.53	5.88
Hex-2: 28 d	10.1	2.05	1.58	0.53	2.24	12.4

**Figure A1:**
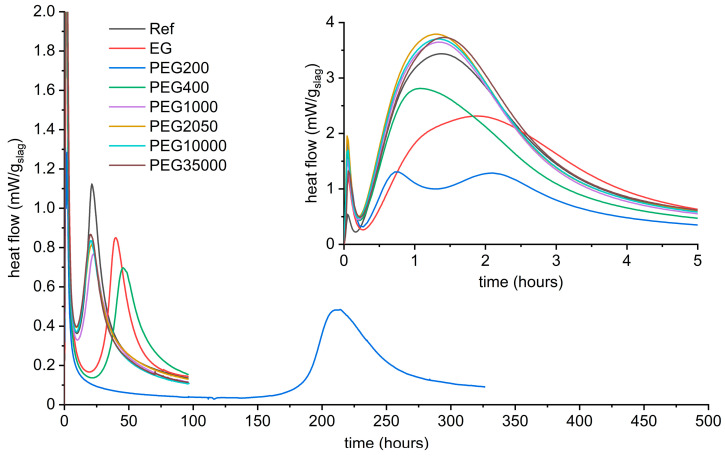
Effect of polyethylene glycol molecular weight on the calorimetric response of waterglass-activated slag.

**Figure A2:**
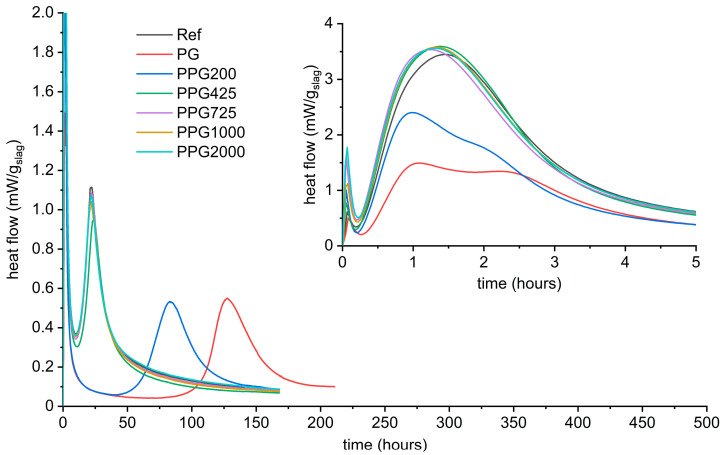
Effect of polypropylene glycol molecular weight on the calorimetric response of waterglass-activated slag.

**Figure A3:**
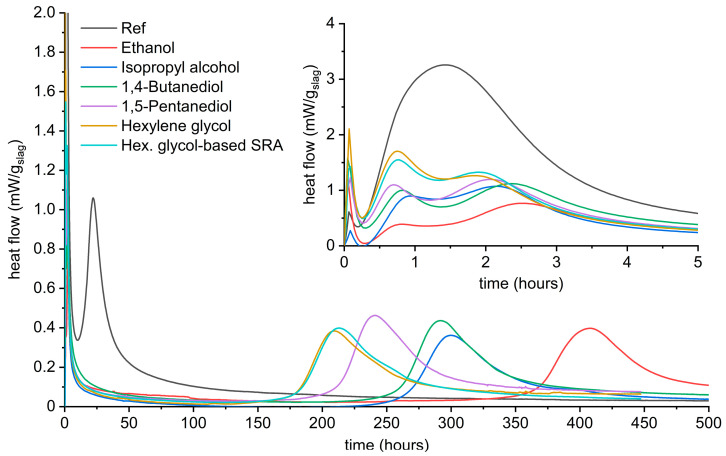
Effect of selected alcohols on the calorimetric response of waterglass-activated slag.

## Data Availability

The data presented in this study are available on request from the corresponding author. The data are not publicly available due to ongoing research.

## Supplementary Materials

The following supporting information can be downloaded at: https://www.mdpi.com/article/10.3390/ma16051908/s1, Video S1: Color change of AAS pastes correlated to its calorimetric response.

## Appendix A. Effect of Various Organic Admixtures on AAS Hydration

In this case, the slag and the activating solution (with 2% admixture, if any) were put separately in the calorimeter and after the thermal equilibration to 25 °C mixed together using glass Admix vials and a built-in Teflon stirrer for 3 min. Time-zero is the first contact of liquids with slag. The results are given in Figure A1, Figure A2 and Figure A3.

Brief summary of results

- Various alcohols can modify the process of alkaline activation of slag.

  → Reduced intensity of the second calorimetric peak and its splitting into two peaks

  → Delayed and more diffuse third calorimetric peak

- Generally: The lower the second peak (or double peak), the more delayed the third peak

Effect of alcohol structure on the delay of the maximum of the third calorimetric peak

- It is hard to find general trends, but some partial trends are observable as follows.
- Number of hydroxyl groups: Monoalcohols are stronger retarders than the corresponding diols

  → ethanol >> ethylene glycol

  → isopropyl alcohol >> propylene glycol

- Length of the carbon chain of diols

  → 1,4-butanediol > 1,5-pentanediol > propylene glycol > ethylene glycol

- Polymerization degree

  → PEG200 > PEG400 > EG > PEG1000, PEG2050, PEG10000, PEG35000 (no delay)

  → PG > PPG200 > PPG 425, PPG725, PPG1000, PPG2000 (no delay)
